# Risk of dementia according to the smoking habit change after ischemic stroke: a nationwide population-based cohort study

**DOI:** 10.1038/s41598-022-27083-0

**Published:** 2022-12-27

**Authors:** Dae Young Cheon, Kyung do Han, Mi Sun Oh, Kyung-Ho Yu, Byung-Chul Lee, Chi-Hun Kim, Yerim Kim, Sang-Hwa Lee, Chulho Kim, Jae-Sung Lim, Minwoo Lee

**Affiliations:** 1 Division of Cardiology, Department of Internal Medicine, Dongtan Sacred Heart Hospital, Hwaseong, South Korea; 2 Department of Statistics and Actuarial Science, Soongsil University, Seoul, South Korea; 3 Department of Neurology, Hallym University Sacred Heart Hospital, Anyang, South Korea; 4 Department of Neurology, Kangdong Sacred Heart Hospital, Seoul, South Korea; 5 Department of Neurology, Chuncheon Sacred Heart Hospital, Chuncheon, South Korea; 6 Department of Neurology, Asan Medical Center, University of Ulsan College of Medicine, 88, Olympic-Ro 43-Gil, Songpa-Gu, Seoul, 05505 South Korea; 7 Department of Neurology, Hallym University College of Medicine, Hallym University Sacred Heart Hospital, Anyang, South Korea

**Keywords:** Dementia, Stroke

## Abstract

There is a paucity of research regarding the association between the risk of incident dementia and changes in smoking habits in the acute ischemic stroke population. We aimed to investigate the effects of smoking habit change on the risk of incident dementia in an ischemic stroke population using data from the Korean National Health Insurance Services Database. This nationwide population-based cohort study included 197,853 patients with ischemic stroke. The patients were divided into never smokers, former smokers, smoking quitters, sustained smokers, and new smokers, based on the 2-year change in smoking status between the two consecutive health examinations before and after the index stroke. The patients were followed up from the index date to 2018 to assess the development of dementia. Dementia was further categorized into Alzheimer’s, vascular, and other types of dementia according to the International Classification of Diseases, Tenth Revision diagnosis. Multivariable Cox proportional hazards models were used to assess the association between changes in smoking habits and the risk of dementia. After a median of 4.04 years of follow-up, 19,595 (9.9%) dementia cases were observed. Among them, 15,189 (7.7%) were diagnosed with Alzheimer’s disease dementia and 2719 (1.4%) were diagnosed with vascular dementia. After adjusting for covariates, including age, sex, alcohol intake habits, cigarette pack-year, regular physical activity, income, history of hypertension, diabetes mellitus, dyslipidemia, and chronic kidney disease, new smokers, sustained smokers, and smoking quitters were significantly associated with a higher risk of all-cause dementia than never smokers (adjusted hazard ratio [aHR] 1.395, 95% confidence interval [CI] 1.254–1.552; aHR 1.324, 95% CI 1.236–1.418; and aHR 1.170, 95% CI 1.074–1.275, respectively). Similar trends were observed for both Alzheimer’s dementia and vascular dementia, but the association between new smokers and vascular dementia was not significant. The impact of smoking habit change was more prominent in the 40–65-year-old group. New and sustained smokers had a substantially higher risk of incident dementia after ischemic stroke than never smokers. Smoking quitters also had an elevated risk of incident dementia, but the detrimental effects were lower than those in new and sustained smokers.

## Introduction

Post-stroke cognitive impairment (PSCI) is one of the major but underrepresented causes of disabilities after ischemic stroke^1^. Although research and clinical practice have mainly focused on functional physical outcomes, cognitive decline is a crucial aspect of post-stroke survivors^2^. More than 20–80% of stroke survivors experience cognitive impairment ranging from mild cognitive impairment to dementia^1,3^. Even minor ischemic stroke (IS)^4^ or transient ischemic attack^5^ may affect activities of daily living and cognitive functions, including executive function and memory, and hinder the return to work.

Despite the clinical significance and burden of post-stroke cognitive impairment, effective disease-modifying treatments are still lacking. While lifestyle modifications, including smoking cessation, alcohol abstinence, and regular physical activity, are associated with a reduced risk of incident dementia^6^ and acute stroke recurrence^7^, the effects of lifestyle modification on post-stroke cognitive impairment have been inconclusive^8,9^. Although previous studies have described a relationship between smoking habits and the risk of incident dementia and stroke in the general population, data on the association between the risk of incident dementia and changes in smoking habits in the acute IS population are scarce^6,10^.

Therefore, we aimed to investigate the impact of smoking habit changes on the risk of incident dementia in an acute IS population using the Korean National Health Insurance Services (NHIS) Database.

## Methods

### Standard protocol approvals, registrations, and patient consents

This study was approved by the Institutional Review Board (IRB) of Dongtan Sacred Heart Hospital (IRB number: HDT 2022-04-002), which waived the need for informed consent owing to the retrospective design and utilization of encrypted patient data. All study methods were performed in accordance with the Declaration of Helsinki.

### Data source and study population

Data were obtained from the Korean NHIS Database^11^. The Korean NHIS is a single-payer mandatory insurance system that provides medical coverage to almost the entire Korean population (approximately 97%). The Korean NHIS has a thorough claims database of participants’ medical usage for review and reimbursement of medical expenses. The database includes demographic variables such as age, sex, International Classification of Diseases, Tenth Revision (ICD-10) diagnoses, health care utilization, prescription records, and insurance type. The Korean NHIS recommends receiving a biannual health checkup for all beneficiaries older than 40 years, without expenses. The health checkup includes medical history, a self-reported lifestyle behavior questionnaire, laboratory tests, and physical examinations. The participation rate in screening examinations was as high as 74.8% in 2014^11^.

We screened participants who were newly diagnosed with IS between January 1, 2010 and December 31, 2016 with a primary diagnosis of IS (ICD-10 code I63–64) and underwent magnetic resonance imaging or computed tomography during hospitalization (n = 1,005,879)^12^. Among them, we only included patients who underwent a national health checkup within 2 years before and after the index IS (n = 264,639) to assess the association between smoking habit changes and dementia development. We excluded participants younger than 40 years of age, those with missing data from the health checkup and smoking-related questionnaires, and those with a previous diagnosis of any type of dementia or stroke. In total, 197,853 patients were included in this study. The case-record form for the collected data from the recruited cohort is demonstrated in Supplemental Table 1.

### Definition of changes in smoking habit

Data regarding smoking habits were collected using a self-reported structured questionnaire at the time of the health checkup within 2 years before and after the index IS. Study participants were categorized according to changes in smoking status before and after the IS diagnosis. In this questionnaire, individuals chose one answer among “never smoked”, “ex-smoker”, and “sustained smoker”. The cumulative amount of smoking in pack-year was also recorded through responses among less than 10, 10–19, 20–39, and 40 or more cigarettes per day. Using these sequential answers, patients were categorized into five groups: never smoker, former smoker, smoking quitter after IS, new smoker, or sustained smoker. Never smokers were defined as patients who never smoked; former smokers as patients who quit smoking before the first health examination and remained non-smokers at the next health examination; smoking quitters after IS as patients who were sustained smokers at the first health examination but became ex-smokers after IS diagnosis at the next health examination; new smokers as patients who were sustained smokers at the second examination but never smokers at the first examination; and sustained smokers as those who were continuously smokers at the first and second health examinations.

### Covariates

We collected covariates from the second general health checkup for each participant. We collected demographic characteristics, including age, sex, weight, height, waist circumference, and vascular risk factors, including previous history of hypertension, diabetes mellitus, dyslipidemia, and chronic kidney disease. Additionally, data on lifestyle behaviors, including physical activity, alcohol consumption history, and income levels, were also collected. Data on physical activity and alcohol consumption were collected using self-report questionnaires. For this analysis, if the average alcohol intake per day (g/day) exceeded 0 g/day, the participants were grouped as alcohol users. Further, participants who exercised moderately > 5 days a week or performed vigorous exercise for > 3 days a week were grouped into the regular physical activity group. Low-income level was defined as the composite of medical benefit beneficiaries and if the patients were included in the lowest quartile of yearly income. Laboratory data, such as a random glucose level, total cholesterol level, glomerular filtration rate according to the Modification of Diet in Renal Disease equation, and systolic/diastolic blood pressure, were also collected. Among these covariates, age, sex, previous history of hypertension, diabetes, dyslipidemia, chronic kidney disease, alcohol consumption, physical activity, income level and cigarette pack-year, which are established risk factors for the development of dementia, were adjusted in the main analysis^6^.

### Study outcomes

The primary outcome of this study was a new diagnosis of all-cause dementia, including Alzheimer disease (AD), vascular dementia (VaD), and other forms of dementia. All-cause dementia was defined using ICD-10 diagnostic codes (F00, F01, F02, F03, G30, or G31) and prescription of anti-dementia medications including donepezil, galantamine, rivastigmine, or memantine. Secondary outcomes included AD dementia (F00 or G30) and VaD (F01) with prescription of the aforementioned anti-dementia medications. When both AD and VaD codes were claimed, the primary diagnosis was selected. When both AD and VaD diagnoses were claimed as additional diagnoses in the index event, we selected the diagnosis according to the primary diagnosis at the next visit. If AD and VaD diagnoses remained as an additional diagnosis during the entire study period, the patients were classified as having other types of dementia. The patients were followed up from the index date at the health screening examination after the diagnosis of IS, until the development of any type of dementia, death, or December 31, 2019, whichever came first.

### Statistical analysis

Baseline and demographic characteristics are presented as numbers (frequencies) for categorical variables and as means ± standard deviations or medians with interquartile ranges for continuous variables. One-way analysis of variance was used to compare continuous variables between each smoking habit change group. For categorical variables, the chi-square test or Fisher exact test was used, as appropriate. The annual event incidence rates were calculated by dividing the total number of dementia diagnoses by the total person-years during the follow-up period (per 1000 person-year [PY]).

Multivariate Cox proportional hazard regression models were used to estimate the association between the smoking habit change and dementia development in patients with IS, and data are presented as adjusted hazard ratios (HRs) and corresponding 95% confidence intervals (CIs) for the primary and secondary outcomes. The never-smoker group was used as the reference group in the main analyses. The Cox models were as follows: model 1 was adjusted for age and sex; model 2 was further adjusted for previous history of hypertension, diabetes, dyslipidemia, chronic kidney disease, alcohol consumption, regular physical activity, and income level; and model 3 was additionally adjusted for cigarette pack-year. Subgroup analyses were conducted according to age group (40–65 years versus 65+ years) and sex. We performed a multivariable Cox proportional hazards regression model for subgroup analyses adjusted for the covariates used in model 4 in the main analysis.

All analyses were 2-tailed and P-values < 0.05 were considered statistically significant. Statistical analyses were performed using SAS (version 9.4; ASA Institute Inc., Cary, NC, USA).

## Results

### Demographic and clinical characteristics of the study population

**Figure 1:**
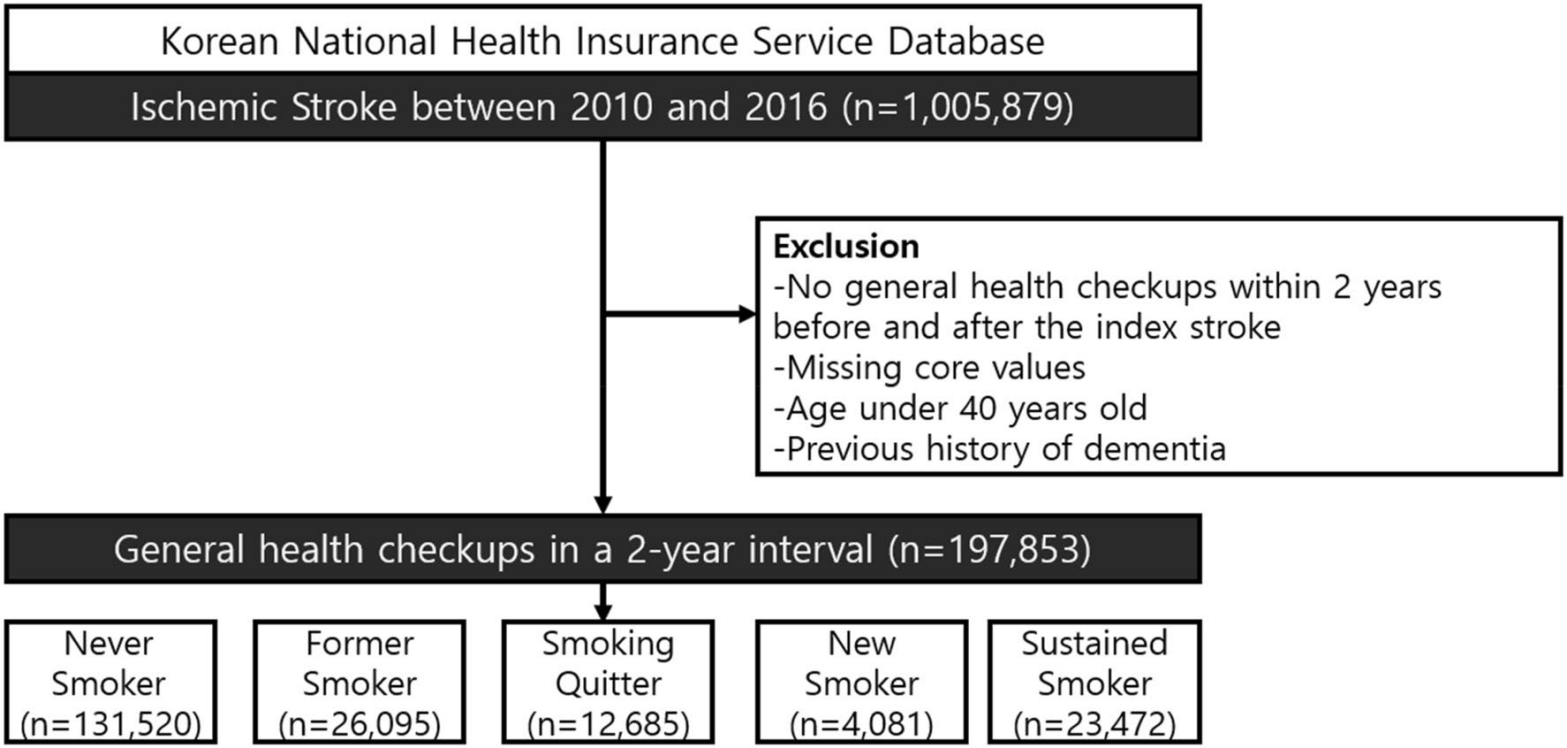
Flowchart of the selection of study subjects.

A total of 1,005,879 patients were diagnosed with acute IS between 2010 and 2016. As our major independent variable for this study was the change in smoking habits, patients who did not receive national health checkups conducted by the NHIS within 2 years before (n = 541,463) and after (n = 199,777) stroke diagnoses were excluded. After excluding patients with missing values and pre-stroke diagnosis of dementia, 197,853 patients with acute IS (mean age, 64.4 ± 10.26 years; men, 46.49%) were finally included in the analysis (Fig. 1).

The participants were categorized into five groups according to the status of smoking habit change, and the baseline demographic and clinical characteristics of each group are shown in Table 1. Patients in the never-smoker group were more likely to be female, older, non-diabetic, and to consume less alcohol than those in the other groups. As patients in the never-smoker group were mostly female (78.04%), their height, weight, and waist circumference were all significantly lower than those in the other groups. Of 36,157 patients who had sustained smoking before the diagnosis of IS, 23,472 (65.0%) continued to smoke even after the diagnosis of IS.

**Table 1:** Demographic and Clinical characteristics according to the smoking habit change in patients with ischemic stroke.

	Never smoker (n = 131,520)	Former smoker (n = 26,095)	Smoking quitter (n = 12,685)	Sustained smoker (n = 23,472)	New smoker (n = 4081)	P-value
Age	65.54 ± 10.19	64.35 ± 10.27	60.92 ± 10.11	60.31 ± 10.15	62.13 ± 10.38	< 0.0001
≥ 65 years	72,458 (55.09)	13,236 (50.72)	4569 (36.02)	7817 (33.3)	1655 (40.55)	< 0.0001
Sex, male	28,877 (21.96)	25,587 (98.05)	12,197 (96.15)	21,848 (93.08)	3463 (84.86)	< 0.0001
Pack years	–	21.36 ± 17.97	29.06 ± 20.13	24.98 ± 16.67	20.00 ± 16.79	–
Alcohol	17,163 (13.05)	12,187 (46.70)	5166 (40.73)	12,998 (55.38)	2103 (51.53)	< 0.0001
Regular PA	24,708 (18.79)	7784 (29.83)	3278 (25.84)	4346 (18.52)	805 (19.73)	< 0.0001
Low income	21,935 (17.13)	3533 (13.87)	2041 (16.51)	4647 (20.22)	824 (20.71)	< 0.0001
Obesity	52,310 (39.77)	10,960 (42.00)	5298 (41.77)	8357 (35.6)	1484 (36.36)	< 0.0001
BMI	24.35 ± 3.19	24.5 ± 2.84	24.46 ± 2.95	23.94 ± 3.15	23.98 ± 3.16	< 0.0001
Waist circumference	81.99 ± 8.70	86.20 ± 7.74	86.02 ± 7.78	84.70 ± 8.27	84.60 ± 8.43	< 0.0001
Diabetes	29,887 (22.72)	7021 (26.91)	3633 (28.64)	6881 (29.32)	1159 (28.4)	< 0.0001
Hypertension	84,543 (64.28)	17,358 (66.52)	8384 (66.09)	14,149 (60.28)	2505 (61.38)	< 0.0001
Dyslipidemia	71,778 (54.58)	14,670 (56.22)	8755 (69.02)	12,514 (53.31)	2064 (50.58)	< 0.0001
CKD	16,812 (12.78)	3025 (11.59)	1370 (10.80)	2106 (8.97)	434 (10.63)	< 0.0001
SBP	12,7.24 ± 15.56	127.43 ± 14.45	126.42 ± 14.59	125.80 ± 15.00	125.52 ± 14.74	< 0.0001
DBP	77.04 ± 9.83	77.46 ± 9.70	77.88 ± 9.85	77.48 ± 9.97	77.13 ± 9.86	< 0.0001
Total cholesterol	186.29 ± 41.32	173.84 ± 39.92	170.16 ± 41.78	179.99 ± 41.90	180.3 ± 41.14	< 0.0001
GFR	83.75 ± 34.98	84.51 ± 56.25	85.84 ± 54.11	88.24 ± 53.46	86.24 ± 50.27	< 0.0001
Glucose	103.83 ± 26.84	106.52 ± 27.22	108.4 ± 31.74	108.86 ± 34.46	107.89 ± 33.38	< 0.0001

*PA* physical activity, *BMI* body mass index, *CKD* chronic kidney disease, *SBP* systolic blood pressure, *DBP* diastolic blood pressure, *GFR* glomerular filtration rate.

### Association of smoking habit change with dementia

During the median 4.04 years (IQR 2.46–5.8), there were 14,562 cases of all-cause dementia, 11,513 cases of AD dementia, and 1839 cases of VaD. All-cause dementia occurred in 14,562 (26.53/1000 PY), 1985 (18.89/1000 PY), 899 (17.26/1000 PY), 397 (23.50/1000 PY), and 1749 (18.14/1000 PY) never smokers, former smokers, smoking quitters, new smokers, and sustained smokers, respectively. The adjusted incidence rates of all-cause dementia were 13.79 PY, 12.95 PY, 16.02 PY, 19.15 PY, and 18.07 PY in the never smoker, former smoker, smoking quitter, new smoker, and sustained smoker groups, respectively.

**Figure 2:**
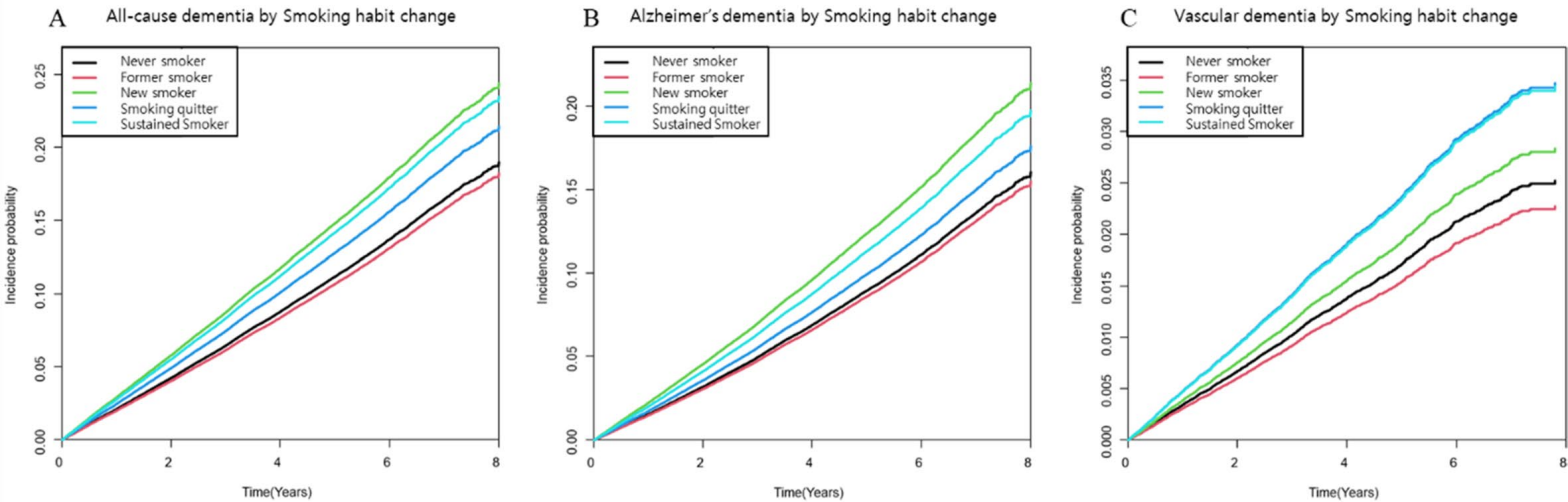
Incidence probability of any dementia ((**A**), P < 0.001), Alzheimer’s dementia ((**B**), P < 0.001), and Vascular Dementia ((**C**), P < 0.001) after ischemic stroke according to the change of smoking habit pattern. *Adjusted for age, sex, alcohol consumption, physical activity, income, diabetes, hypertension, dyslipidemia, chronic kidney disease, and pack years.

The crude incidence rate, crude hazard ratio, and aHR according to the prespecified models for all-cause dementia, AD dementia, and VaD for each group are shown in Table 2. In the multivariate Cox proportional hazard regression analysis, when compared to the never-smoker group, the smoking quitter, sustained smoker, and new smoker groups were significantly associated with the development of all-cause dementia (aHR 1.17, 95% CI: 1.07–1.28; aHR 1.32 95% CI 1.24–1.42; and aHR 1.395 95% CI 1.254–1.552, respectively) Similar associations with the smoking habit change were observed in both secondary outcomes, AD dementia and VaD. However, the new smoker group was not significantly associated with a higher risk of VaD development (aHR 1.126 95% CI 0.842–1.51). Kaplan–Meier curves for the primary and secondary outcomes adjusted for age, sex, regular physical activity, alcohol consumption, income, history of hypertension, diabetes mellitus, dyslipidemia, chronic kidney disease, and cigarette pack-year demonstrated significant differences in primary and secondary outcomes between the groups according to the status of smoking habit changes (Fig. 2).

**Table 2:** Risk for All-cause dementia, AD dementia and VaD according to the smoking habit change.

	n	Event (n)	aIR (%)	aHR_Model 1	aHR_Model 2	aHR_Model 3
**All-cause dementia**						
Never smoker	131,520	14,562	13.79	1 (Ref.)	1 (Ref.)	1 (Ref.)
Former smoker	26,095	1985	12.95	0.926 (0.876, 0.978)	0.952 (0.900, 1.006)	0.949 (0.889, 1.012)
Smoking quitter	12,685	899	16.02	1.177 (1.094, 1.267)	1.176 (1.093, 1.266)	1.170 (1.074, 1.275)
Sustained smoker	23,472	1749	18.07	1.312 (1.240, 1.389)	1.329 (1.255, 1.408)	1.324 (1.236, 1.418)
New smoker	4081	397	19.15	1.377 (1.243, 1.525)	1.399 (1.262, 1.551)	1.395 (1.254, 1.552)
**Alzheimer’s dementia**						
Never smoker	131,520	11,513	10.07	1 (Ref.)	1 (Ref.)	1 (Ref.)
Former smoker	26,095	1480	9.49	0.924 (0.867, 0.985)	0.951 (0.892, 1.015)	0.955 (0.886, 1.029)
Smoking quitter	12,685	629	11.23	1.117 (1.024, 1.218)	1.120 (1.026, 1.222)	1.125 (1.017, 1.246)
Sustained smoker	23,472	1259	12.99	1.282 (1.200, 1.370)	1.301 (1.217, 1.391)	1.306 (1.206, 1.414)
New smoker	4081	308	14.54	1.425 (1.268, 1.601)	1.448 (1.289, 1.628)	1.453 (1.287, 1.641)
**Vascular dementia**						
Never smoker	131,520	1839	2.32	1 (Ref.)	1 (Ref.)	1 (Ref.)
Former smoker	26,095	312	2.09	0.919 (0.798, 1.058)	0.946 (0.821, 1.090)	0.90 (0.765, 1.059)
Smoking quitter	12,685	195	3.22	1.514 (1.283, 1.786)	1.487 (1.259, 1.755)	1.389 (1.140, 1.693)
Sustained smoker	23,472	321	3.18	1.422 (1.238, 1.633)	1.456 (1.265, 1.675)	1.375 (1.164, 1.625)
New smoker	4081	52	2.61	1.142 (0.861, 1.515)	1.179 (0.888, 1.566)	1.127 (0.842, 1.510)

*HR* hazard ratio, *aHR* adjusted hazard ratio, *aIR* adjusted incidence rate, *PY* person-years.

Model 1: age and sex-adjusted, Model 2: age, sex, alcohol consumption, regular physical activity, low-income, diabetes, hypertension, lipid and chronic kidney disease, Model 3: Model 2+ Cigarette pack years.

### Subgroup analyses

To determine whether the association between smoking habit changes and the risk of all-cause dementia was modified by prespecified subgroups, we stratified the patients according to their age (40–65 years versus 65+ years) and sex. In the subgroup analysis, the detrimental effects of new smokers, smoking quitters, and sustained smokers were more prominent in the younger age group than in the older age group. Furthermore, former smokers were significantly associated with a reduced risk of all-cause dementia than the never smokers in the younger patient group. However, the subgroup analysis according to sex showed findings consistent with those of the main analysis (Table 3).

**Table 3:** Subgroup analyses according to the age and sex group and risk for all-cause dementia.

	Smoking habit change	n	All-cause dementia	aIR	aHR_Model 1	aHR_Model 2	aHR_Model 3
Male	Never smoker	28,877	3112	11.91	1 (Ref.)	1 (Ref.)	1 (Ref.)
	Former smoker	25,587	1941	11.00	0.910 (0.860, 0.963)	0.937 (0.885, 0.992)	0.927 (0.867, 0.992)
	Smoking quitter	12,197	844	13.45	1.149 (1.064, 1.240)	1.147 (1.062, 1.238)	1.131 (1.033, 1.238)
	Sustained smoker	21,848	1581	15.13	1.285 (1.209, 1.366)	1.306 (1.227, 1.389)	1.290 (1.198, 1.389)
	New smoker	3463	326	15.94	1.336 (1.192, 1.497)	1.360 (1.213, 1.525)	1.347 (1.195, 1.517)
Female	Never smoker	102,643	11,450	15.92	1 (Ref.)	1 (Ref.)	1 (Ref.)
	Former smoker	508	44	17.11	1.173 (0.872, 1.577)	1.179 (0.877, 1.586)	1.174 (0.873, 1.579)
	Smoking quitter	488	55	20.78	1.451 (1.113, 1.891)	1.482 (1.137, 1.932)	1.473 (1.129, 1.921)
	Sustained smoker	1624	168	20.27	1.423 (1.222, 1.658)	1.415 (1.215, 1.648)	1.406 (1.206, 1.640)
	New smoker	618	71	21.84	1.505 (1.192, 1.901)	1.523 (1.206, 1.924)	1.515 (1.199, 1.915)
P for interaction					0.1243	0.1495	0.1324
40–64 years	Never smoker	59,062	1500	3.46	1 (Ref.)	1 (Ref.)	1 (Ref.)
	Former smoker	12,859	214	2.86	0.827 (0.714, 0.957)	0.843 (0.728, 0.977)	0.843 (0.726, 0.979)
	Smoking quitter	8116	231	5.10	1.542 (1.338, 1.777)	1.506 (1.307, 1.736)	1.506 (1.299, 1.745)
	Sustained smoker	15,655	436	5.34	1.543 (1.382, 1.723)	1.529 (1.368, 1.709)	1.529 (1.361, 1.718)
	New smoker	2426	85	6.16	1.777 (1.426, 2.214)	1.774 (1.424, 2.212)	1.774 (1.421, 2.215)
≥ 65 years	Never smoker	72,458	13,062	39.45	1 (Ref.)	1 (Ref.)	1 (Ref.)
	Former smoker	13,236	1771	38.00	0.942 (0.889, 0.998)	0.970 (0.915, 1.027)	0.969 (0.907, 1.036)
	Smoking quitter	4569	668	43.55	1.103 (1.016, 1.198)	1.107 (1.019, 1.203)	1.107 (1.007, 1.217)
	Sustained smoker	7817	1313	50.18	1.271 (1.194, 1.354)	1.292 (1.213, 1.377)	1.292 (1.200, 1.391)
	New smoker	1655	312	52.36	1.313 (1.170, 1.472)	1.336 (1.191, 1.498)	1.335 (1.186, 1.504)
P for interaction					< 0.0001	< 0.0001	< 0.0001

*HR* hazard ratio, *aHR* adjusted hazard ratio, *IR* incidence rate; *PY* person-years.

## Discussion

In this nationwide, population-based, large-scale cohort study, we evaluated whether changes in smoking status were associated with the risk of dementia in patients with acute IS. Our primary findings were as follows: (1) 65% of patients continued to smoke even after experiencing acute IS; (2) the risk of incident dementia among new smokers, sustained smokers, and smoking quitters was significantly elevated compared with never smokers among those with acute IS; and (3) the detrimental effects of sustained and new smoking on incident dementia were consistent and more prominently elevated in the younger age group than in the older age group. This study is clinically significant in that it confirmed the effect of changes in smoking habits on the occurrence of cognitive impairment after stroke.

When patients experience IS, they are usually recommended to quit smoking and are sometimes aided by nicotine treatment. However, we found that the smoking rate decreased from 18.3 to 11.9% after IS diagnosis. Furthermore, 2% of patients started to smoke even after a diagnosis of IS. As smoking is associated with recurrent stroke^13^ and our findings imply that smoking increases the risk of dementia development after IS, education and active treatment of smoking are of clinical importance.

In previous studies, sustained smokers showed a 38% increased risk of VaD compared with never smokers^14^. Our findings are in line with those studies’ results in that those who continued or initiated smoking had an increased risk of incident dementia in the stroke population. Additionally, we showed that smoking history was associated with a higher risk of incident dementia after stroke. A previous meta-analysis showed that former smoking was not associated with an increased risk of all-cause dementia, AD dementia, or VaD in the general population^15^. Our results imply that, at least in the acute IS population, a history of former smoking may affect the development of incident dementia. In stroke population studies, a multinational post-stroke cognitive impairment consortium revealed that smoking is negatively associated with memory function and perceptual-motor performance, implying that the effects of smoking are likely to be domain-specific^16^. However, a meta-analysis that included six studies regarding the effects of smoking on stroke revealed that smoking was associated with a decreased risk of dementia, with an odds ratio of 0.5 (95% CI 0.3–0.7, P = 0.05). Thus, the effects of smoking on the incidence of dementia in the stroke population have been inconsistent. One of the reasons for the observed inconsistent results in the stroke population, unlike in the general population, may be the collider bias that is often encountered when assessing cognitive outcomes in the stroke population. As both stroke and cognitive impairment may hinder the study enrollment process, which requires a mandatory ambulatory visit, like in our study, the associations between them may be biased.

The potential mechanisms for the association between smoking status and incident dementia have mostly been studied in the general population. Smoking is believed to damage the neurovascular system and cause oxidative stress, inflammation, lipid modification, impaired endothelial function, and acceleration of an atherothrombotic process, which may directly increase the risk of dementia and stroke^17,18^. Furthermore, smoking increases the risk of other cardiovascular risk factors including hypertension^18^ and diabetes mellitus^19^, which are shared risk factors for both dementia and stroke. Thus, the risk of dementia in the acute stroke population may share these potential mechanisms of smoking habits.

Although the 2020 report of the Lancet Commission reported that smoking is an established risk factor for dementia in later life (age > 65 years) but not in midlife (age < 65 years)^6^, our subgroup analysis revealed that the younger age group showed a more prominent risk than the older age group. As smoking is associated with a higher risk of mortality and morbidity in the stroke population, specifically in later life, competing risks, including all-cause death in the older age group, may be the potential speculations for this finding. Furthermore, female patients who continued or initiated smoking tended to have a higher risk of developing all-cause dementia, AD dementia, and VaD. This finding is in line with previous studies showing a higher risk of dementia in the general female population^20^. Female smokers are more vulnerable to cardiovascular disease, which may increase the risk of incident dementia in the stroke population^21^.

For secondary outcomes, we investigated the impact of changes in smoking habits on AD dementia and VaD. The association between these factors was mostly consistent with the results of the primary outcome analysis. Although new smokers had the highest risk of developing AD dementia, they were not associated with an increased risk of VaD. Although speculative, patients with IS who were healthy enough and functionally independent may have been the majority of the new smoker group, and the short-term follow-up of patients (median 4.14 years) may have contributed to these findings.

This study has a couple of limitations. First, given the nature of a population-based cohort study based on claims data, this stroke population data lacked important clinical variables, including stroke severity, discharge antithrombotics, discharge statins, and history of atrial fibrillation, which are established risk factors for dementia after stroke. Furthermore, as our stroke population only included those who were able to attend an ambulatory visit to national health checkups and undergo structured questionnaires on their own, our study population was expected to have IS of mild severity. Thus, our findings preclude generalization due to severity issues. Second, the smoking habit assessment was solely conducted according to the patient’s self-reporting without chemical verification of their smoking status. Third, the diagnosis of dementia was dependent on ICD-10 diagnostic codes and the prescription of anti-dementia drugs. Thus, the validity of the diagnosis of the dementia subtype may be less accurate. Despite these limitations, this study has several strengths. First, this large nationwide cohort with two consecutive structured questionnaires enabled us to determine the effects of smoking habit changes before and after the index stroke diagnosis on incident dementia in real-world practice. Second, the number of patients who sustained or initiated smoking after the diagnosis of IS was determined.

In conclusion, new smokers sustained smokers, and smoking quitters have a significantly higher risk of incident dementia after IS than never and former smokers. Our study’s results also demonstrated that the detrimental effect of smoking was further increased in the younger age group than in the older age group. To this end, our results have crucial clinical implications, specifically suggesting that stroke physicians should actively advise patients to stop smoking, not just to prevent stroke recurrence, but also to reduce the risk of further cognitive impairment.

## Supplementary Information

Supplementary Table 1.

## Data Availability

Anonymized dataset for this study is publicly available from the Korean National Health Insurance Sharing Service and can be accessed at https://nhiss.nhis.or.kr/bd/ab/bdaba000eng.do.
