# Supplementary material for: Risk of dementia according to the smoking habit change after ischemic stroke: a nationwide population-based cohort study
Source: Sci Rep. 2022 Dec 27;12:22422. doi: 10.1038/s41598-022-27083-0 (PMC9794689; doi:10.1038/s41598-022-27083-0)
Supplement: Supplementary file 1 — Supplementary Table 1. [file 41598_2022_27083_MOESM1_ESM.docx]

**Supplemental Table 1. The case report form for the study cohort**

| **Case Report Form** |
| --- |
| **Risk of Dementia According to the Smoking Habit Change after Ischemic Stroke: A Nationwide Population-Based Cohort Study** |

| **Inclusion Criteria** | | | |
| --- | --- | --- | --- |
|  |  | **YES** | **NO** |
| **1.** | **Diagnosis of Ischemic stroke [ICD 10 I63-64]** | **□** | **□** |
| **2.** | **Received two consecutive national health check-ups before and after**  **the diagnosis of stroke** | **□** | **□** |
|  | | | |

| **Exclusion Criteria** | | | |
| --- | --- | --- | --- |
|  |  | **YES** | **NO** |
| **1.** | **Age less than 40 years** | **□** | **□** |
| **2.** | **Missing data on smoking status in the national health check ups.** | **□** | **□** |
| **3.** | **Previous diagnosis of ischemic stroke or any types of dementia** | **□** | **□** |
|  | | | |

| **Demographic features of subjects** | | | |
| --- | --- | --- | --- |
| **Demographic features of subjects** | | | |
| **Age** |  | **Education** | **Years** |
| **Sex:** | **□ M □ F** | **Height**  **Weight**  **Waist circumference** | **cm**  **Kg**  **cm** |
|  |  | | |

| **Risk factors** | |  |
| --- | --- | --- |
| **Hypertension** | **□No** | **□ Yes** |
| **Diabetes** | **□No** | **□ Yes** |
| **Dyslipidemia** | **□No** | **□ Yes** |
| **Coronary artery disease** | **□No** | **□ Yes** |
| **Atrial fibrillation** | **□No** | **□ Yes** |
| **Chronic Kidney Disease** | **□No** | **□ Yes** |
| **Smoking status**  **1^st^ Questionnaire** | **□ No** | **□ Yes** |
|  | **□ Never**  **□ Former** | **□ less than 10/day**  **□ 10-19 per day**  **□ 20-39 per day**  **□40 or more per day** |
| **Smoking status**  **2^nd^ Questionnaire** | **□ No** | **□ Yes** |
|  | **□ Never**  **□ Former** | **□ less than 10/day**  **□ 10-19 per day**  **□ 20-39 per day**  **□40 or more per day** |
| **Alcohol intake** | **□ No** | **□ Yes(Amount : /per day)** |
| **Regular Physical activity** | **□ No** | **□ Yes** |
| **Income level** | **□ Low level** | **□ Otherwise** |
| **Previous stroke/TIA history** | **□No** | **□Yes** |

| **Laboratory findings** |  |
| --- | --- |
| **Random glucose level** | mg/dL |
| **Total cholesterol level** | mg/dL |
| **Glomerular filtration rate** |  |
| **Systolic blood pressure** | mmHg |
| **Diastolic blood pressure** | mmHg |

| **Final Diagnosis** |  |
| --- | --- |
| **Alzheimer’s dementia** |  |
| **Vascular Dementia** |  |
| **Other dementia** |  |
